# Kinect-based choice reaching and stepping reaction time tests for clinical and in-home assessment of fall risk in older people: a prospective study

**DOI:** 10.1186/s11556-016-0162-2

**Published:** 2016-01-30

**Authors:** Andreas Ejupi, Yves J. Gschwind, Matthew Brodie, Wolfgang L. Zagler, Stephen R. Lord, Kim Delbaere

**Affiliations:** Assistive Healthcare Information Technology Group, Austrian Institute of Technology, Vienna, Austria; Vienna University of Technology, Vienna, Austria; Neuroscience Research Australia, University of New South Wales, Sydney, Australia

**Keywords:** Older adults, Accidental falls, Fall risk assessments, Microsoft kinect, Sensors, Reaction time, Reaching, Stepping

## Abstract

**Background:**

Quick protective reactions such as reaching or stepping are important to avoid a fall or minimize injuries. We developed Kinect-based choice reaching and stepping reaction time tests (Kinect-based CRTs) and evaluated their ability to differentiate between older fallers and non-fallers and the feasibility of administering them at home.

**Methods:**

A total of 94 community-dwelling older people were assessed on the Kinect-based CRTs in the laboratory and were followed-up for falls for 6 months. Additionally, a subgroup (*n* = 20) conducted the Kinect-based CRTs at home. Signal processing algorithms were developed to extract features for reaction, movement and the total time from the Kinect skeleton data.

**Results:**

Nineteen participants (20.2 %) reported a fall in the 6 months following the assessment. The reaction time (*fallers:* 797 ± 136 ms, *non-fallers:* 714 ± 89 ms), movement time (*fallers:* 392 ± 50 ms, *non-fallers:* 358 ± 51 ms) and total time (*fallers:* 1189 ± 170 ms, *non-fallers:* 1072 ± 109 ms) of the reaching reaction time test differentiated well between the fallers and non-fallers. The stepping reaction time test did not significantly discriminate between the two groups in the prospective study. The correlations between the laboratory and in-home assessments were 0.689 for the reaching reaction time and 0.860 for stepping reaction time.

**Conclusion:**

The study findings indicate that the Kinect-based CRT tests are feasible to administer in clinical and in-home settings, and thus represents an important step towards the development of sensor-based fall risk self-assessments. With further validation, the assessments may prove useful as a fall risk screen and home-based assessment measures for monitoring changes over time and effects of fall prevention interventions.

## Background

Falls are an important problem in older people and a major public health issue. A range of physiological impairments including poor balance [1, 2], impaired gait [3, 4], muscle weakness [5, 6] and slow voluntary reaction times [7–9] have been associated with falls.

Quick protective reactions that involve reaching or stepping movements are important to avoid falls [10] or to reduce the risk of severe injuries [11, 12]. Tests that reveal deficits in upper- or lower-limb responses may help identify older people at risk of falls or increased risk of fall injury because of poor protective responses after a loss of balance has occurred [10, 13].

Previously, simple and choice reaction times with finger- or foot-press responses have been measured with electronic timers and switches in the laboratory [7, 9]. Long term monitoring by regularly repeated assessments would allow identifying the risk of falling over time or the tracking of improvements following an exercise program. But, because of limited resources of most healthcare systems, regularly repeated assessments are not feasible. Inexpensive, easy to administer, portable and accurate reaction time tests would make it possible to incorporate these tests in clinical practice or even self-assessments performed at home.

Recent advances in sensor technologies and research in human computer interaction hold great promise for these new methods of fall risk assessment [14, 15]. However, to date, only a few studies have focussed on the development of a home-based reaction time test for fall risk assessment. In one study a choice reaction time test using an infrared laser in combination with a plus-shaped mat was developed to measure stepping responses to optical cues [16]. Another stepping study evaluated a mat-based system with pressure sensors to assess and improve stepping responses [17, 18]. To our knowledge, no research exists on home-based upper-limb reaction time tests.

The aim of this study was to examine the potential of Kinect-based, portable and low-cost assessment tests of choice reaching and stepping reaction time (referred as Kinect-based CRTs) to assess fall risk. The main aims of the study were to: (i) investigate whether the Kinect-based CRTs could differentiate between older fallers and non-fallers and (ii) examine the feasibility of conducting the CRT tests in a home setting.

## Methods

### Participants

A total of 94 community-dwelling older people living in retirement villages in Sydney, Australia participated in this study. The sample was drawn from two trials: 1) iStoppFalls trial (ACTRN12614000096651) [19] and 2) SureStep trial (ACTRN12613000671763). The inclusion criteria were: living independently (i.e. not in assisted living or nursing homes), aged 65 years or older and being ambulant with or without the use of a walking aid. The exclusion criteria were: medically unstable, suffering from major cognitive impairment (Mini-Cog < 3), neurodegenerative disease or colour blindness. Written informed consent was obtained from all participants prior to data collection. The study was approved by the University of New South Wales Human Studies Ethics Committee.

### Kinect-based choice reaction time tests (Kinect-based CRTs)

**Fig. 1:**
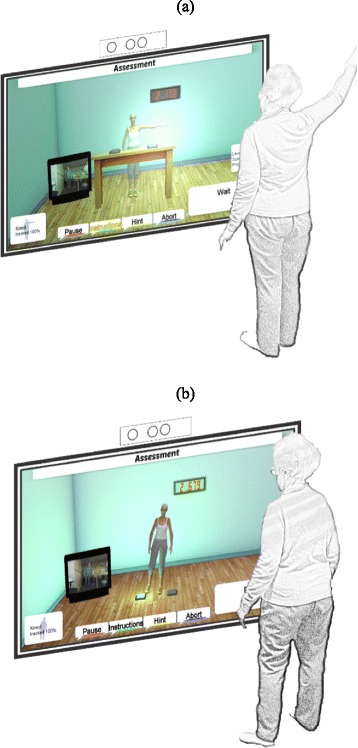
Schematic representations of Kinect-based CRT tests: **a**) reaching reaction time test and **b**) stepping reaction time test

**Fig. 2:**
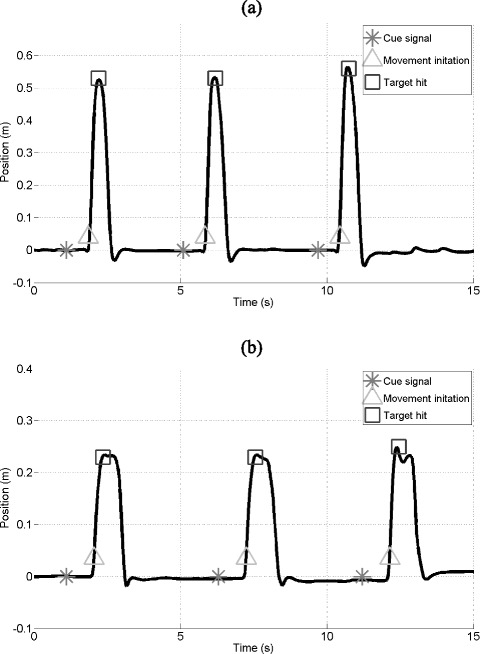
Skeleton data of **a**) hand tracking (reaching reaction time test) and **b**) foot tracking (stepping reaction time test) of the Microsoft Kinect. The figures illustrate three responses of the hand (**a**) and foot (**b**) to cue signals

The Kinect-based CRTs are tests which rely on video-based motion capture technology (i.e. Microsoft Kinect). They comprise 1) a choice reaching reaction time test (Fig. 1a) and 2) a choice stepping reaction time test (Fig. 1b). When conducting the Kinect-based CRT tests participants see themselves represented as an avatar in a virtual environment on a TV screen. The tests start with the participant standing in a normal comfortable position with the arms by the side. Two lights, one to the left and one to the right side of the avatar, flash up in random order. In the reaching reaction time test, participants are instructed to lift their corresponding arm to the flashing light as fast as possible. In the stepping reaction time test, participants have to take a step onto the flashing light, using the left foot when the left light flashes and the right foot when the right light flashes, as quickly as possible.

### Protocol

The study protocol included the following parts:

1. *Laboratory assessment:* All participants were assessed on the Kinect-based CRTs and on clinical tests for reaction time and fall risk.
2. *Association with future falls:* Participants were followed up for falls for 6 months and the ability of the Kinect-based CRT tests to differentiate between the fallers and non-fallers was investigated.
3. *In-home assessment:* Following the laboratory assessment the Kinect-based CRTs were conducted with a subgroup of participants at home and the relationships between the laboratory and in-home assessments were analysed.

### Laboratory assessment

All participants were initially assessed with the Kinect-based CRT tests in the laboratory. For each participant 40 reaching and stepping responses were recorded with a short break of less than a minute after 20 responses. The first five trials were practice trials and excluded from data analysis. The assessments were video recorded with two video cameras (i.e. front and side view) to support the researchers during the data analysis process. The Physiological Profile Assessment (PPA) was conducted as an estimate of the overall fall risk of the participants. The PPA is based on tests which assess sensorimotor abilities: balance (sway when standing on medium-density foam with eyes open), lower extremity muscle strength (knee extension), contrast sensitivity (Melbourne edge test (MET), peripheral sensation (proprioception) and single hand (i.e. finger-press) reaction time [9].

The convergent validity of the Kinect-based CRT tests in relation to the simple reaction time of the PPA and choice reaction time of the Attention Network Test (ANT) were examined. The ANT is a computer-based test where participants had to determine whether a central arrow points to the left or right and to press the corresponding button on a PC-keyboard as quickly as possible [20].

### Association with future falls

Participants were followed-up for 6 months and asked to report their falls with monthly falls calendars. Follow-up telephone interviews were conducted if participants failed to return their calendars. A fall was defined as ‘an unexpected event in which the person comes to rest on the ground, floor, or lower level’ [21]. Participants were classified as fallers if they experienced at least one fall in the 6 months follow-up period.

### In-home assessment

The feasibility to administer the Kinect-based CRTs at home was examined in a subsample of 20 participants. The system was installed in the participants’ homes and the CRT tests were conducted under supervision of a trained researcher. The time gap between the laboratory assessment and the in-home assessment was on average 40 (±20) days.

### Data acquisition and analysis

The Microsoft Kinect is a marker-free computer vision sensor that can measure three-dimensional motion of a person. In the laboratory, the Kinect sensor was placed in front of the TV screen at a height of 80 cm and a distance of 200 cm from the participants. Skeleton data of anatomical landmarks in world coordinates were recorded using the Kinect Software Development Kit for Windows with a sampling rate of 30 Hz and a resolution of 640 × 480 pixels.

For the Kinect-based CRTs the horizontal displacement data (i.e. movements in the x-axis to the left or the right) of the Microsoft Kinect sensor were used for the algorithms. In detail, the skeleton data of the left and right hand tracking were obtained for the reaching reaction time test and the tracking data of the feet for the stepping reaction time test. The signals were low-pass filtered using a 4^th^ order Butterworth filter with a cut-off frequency of 2 Hz to reduce noise. The following features were automatically extracted from each recording (Fig. 2):

1. *Reaction time:* The reaction time was defined as the time from the cue signal until the first movement of the hand or foot. The movement initiation was detected as a change in position of at least 5 cm (i.e. to the left or right) compared to the rest position. The mean across all reaction times was calculated.
2. *Movement time:* The movement time was defined as the time from the movement initiation until the corresponding virtual target was hit by the hand or foot. Incorrect movements for example in the opposite direction of the cue signal were excluded. The mean movement time across all correctly identified movements was calculated.
3. *Total time:* The total time was defined as the sum of the reaction and movement time.

### Statistical analysis

One-way ANOVA was used to evaluate mean differences in the test measures between the fallers and non-fallers. Pearson’s correlation coefficients were calculated to quantify convergent validity and the relationship between the laboratory and in-home assessments. Correlation results were categorized as weak (0.1 – 0.3), moderate (0.4 – 0.6), and strong (0.7 – 0.9) [22]. P-values less of 0.05 were considered to be statistically significant. Signal processing, data analysis and statistical analysis were performed in MATLAB 8.2 (R2013b).

## Results

Ninety-four persons (62 women) aged 80.6 ± 6.9 years participated in the study. On average, participants were 163.7 ± 9.8 cm tall, weighed 72 ± 15.2 kg, had a Body-Mass-Index (BMI) of 26.8 ± 4.7 and PPA fall risk score of 1.48 ± 0.88 indicating a moderate risk of falls [9].

### Convergent validity

The Kinect-based reaching reaction time was significantly correlated with the simple reaction time of the PPA (*r* = 0.338, *p* < 0.001) and choice reaction time of the ANT (*r* = 0.593, *p* < 0.001). Similarly, the Kinect-based stepping reaction time measurement was significantly correlated to the PPA reaction time (*r* = 0.403, *p* < 0.001) and ANT reaction time (*r* = 0.576, *p* < 0.001) tests.

### Association with future falls

Nineteen participants (20.2 %) reported one or more falls in the 6 months following the assessment. There was no significant difference in age, height, weight or BMI between the fallers and non-fallers. Fallers were significantly slower than non-fallers on the reaching reaction time test measurements (Table 1). The stepping reaction time test, the simple reaction time of the PPA assessment and ANT choice reaction time did not significantly discriminate between the groups.

**Table 1:** Test scores (mean ± standard deviation) of the Kinect-based CRT tests and clinical reaction time measurements for the fallers and non-fallers

Measurement	Fallers (*n* = 19)	Non-fallers (*n* = 75)	*P*-Value
Choice reaching reaction time test			
Reaction time (ms)	797 ± 136	714 ± 89	0.002**
Movement time (ms)	392 ± 50	358 ± 51	0.010*
Total time (ms)	1189 ± 170	1072 ± 109	<0.001**
Choice stepping reaction time test			
Reaction time (ms)	894 ± 168	849 ± 150	0.257
Movement time (ms)	360 ± 55	342 ± 44	0.125
Total time (ms)	1254 ± 189	1190 ± 158	0.132
Clinical measurements			
ANT choice reaction time (ms)	827 ± 118	785 ± 141	0.232
PPA simple reaction time (ms)	243 ± 32	235 ± 42	0.398

* *P* < 0.05, ** *P* < 0.01, *ANT* Attention Network Test, *PPA* Physiological Profile Assessment

### In-home assessment

**Fig. 3:**
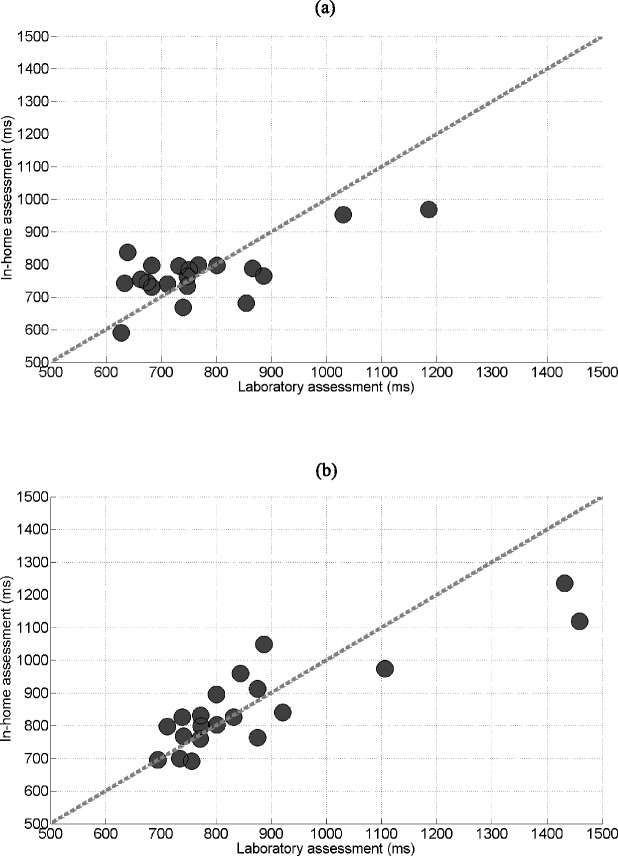
Correlations between the results from the laboratory and in-home assessments of the (**a**) reaching reaction time test (*r* = 0.689) and (**b**) stepping reaction time test (*r* = 0.860)

The in-home assessments were conducted with 20 participants (14 women, 2 fallers). Figure 3 illustrates the linear relationships between the laboratory and in-home assessments. On average, the Kinect-based reaching reaction time was 772 ms ± 85 ms at home compared with 771 ms ± 139 ms in the laboratory (*p* = 0.987). The correlations were moderate to strong for the reaction time (*r* = 0.689, *p* < 0.001), movement time (*r* = 0.505, *p* = 0.023) and total time (*r* = 0.737, *p* < 0.001). Similarly, there was no significant difference between the Kinect-based stepping reaction time at home with 862 ms ± 144 ms and in the laboratory with 876 ms ± 215 ms (*p* = 0.594). The correlations were strong for the reaction time (*r* = 0.860, *p* < 0.001) and total time (*r* = 0.814, *p* < 0.001). Movement time was moderately correlated (*r* = 0.609. *p* = 0.004).

## Discussion

This study, examined the feasibility of Kinect-based reaching and stepping reaction time tests. Signal processing algorithms were developed to quantify performance on these tests and the convergent and discriminant validity of the derived sensor-based measurements were evaluated. To our knowledge, this is the first study using a Kinect-based approach to assess upper- and lower-limb reaction time conducted in both the laboratory and home in community-dwelling older people.

We found that fallers were slower than non-fallers on the reaching reaction time test measurements. Our finding is consistent with the results of previous studies showing that slow reactions are associated with an increased risk of falling [10, 23]. Use of the upper-limbs is a common response to prevent a fall and to reduce risk of injuries [10, 11]. It has recently been reported that in frail older people the protective responses are often ineffective because of lack of strength and movement speed [13]. The Kinect-based CRT may help to reveal deficits in upper-limb responses for targeted improvement.

In a previous study, a test of stepping reaction time was shown to discriminate well between older fallers and non-fallers based on 12 month fall history [24], but this could not be verified in this prospective study, possibly because of the short follow-up period. We believe the Kinect-based stepping reaction time test has some benefits when compared to other approaches. Other systems require a step mat placed on the floor. This could be seen as an advantage as it provides physical targets during stepping. However, step mats also pose a potential trip hazard, require more time to set up and limit movements to the pre-defined fields.

The reaction times measured with the Kinect-based CRTs were on average longer compared to measurements from traditional tests using electronic timers and switches [7, 9]. However, when compared the results to studies using similar technical equipment the reaction times were almost identical [16, 17]. This can be explained with a delay of the TV and Kinect sensor for data acquisition, video processing and display. Future studies are warranted to investigate the relevance of this possible measurement error.

The advantages of a Kinect-based system are 1) easy to set up - no further physical equipment is needed, 2) safe - no additional trip hazards, 3) inexpensive - the Microsoft Kinect is a widely available consumer device and 4) fairly accurate [25, 26] - enables whole body tracking of participants’ movements. These characteristics enable its use in a clinical setting or even in the homes of the older people as an assessment or training tool. Currently, regular repeated assessments are not feasible in clinical practice and therefore the assessments are often only weakly associated with falls. The correlations between the Kinect-based CRT laboratory and in-home assessments were moderate to strong which suggests the conduct of the Kinect-based CRT tests at home is feasible. Noteworthy, the correlations were stronger for the reaction times compared to the movement times. This could indicate that the reaction time measurements are less dependent on the environmental conditions (e.g. distance to the Kinect sensor, size of TV).

We acknowledge certain study limitations. The in-home assessment was conducted with a relatively small subgroup of participants and that might limits the generalizability of the results. Furthermore, for organisational reasons, all 94 participants had to be tested in the laboratory first, before the Kinect-based CRTs could be conducted in the private homes. This resulted in a longer time gap between the laboratory and home assessments for people who were assessed early compared to people who were assessed later in the study. The sample size was only moderate for a fall risk study and the prospective follow-up period relatively short for capturing sufficient fall events. Larger-scale studies are therefore necessary to confirm the presented results. However, our encouraging findings suggest the Kinect-based CRTs could be conducted by the following means 1) tests performed in a clinical setting, 2) tests administered in regular home visits by trained personnel or 3) tests performed independently and unsupervised as self-assessments.

## Conclusions

In summary, our findings indicate the Kinect-based CRT tests are feasible to administer in clinical and in-home settings, and thus represents an important step towards the development of sensor-based fall risk self-assessments. With further validation, the assessments may prove useful as a fall risk screen and home-based assessment measures of upper- and lower-limb movements for monitoring changes over time as well as the effects of fall prevention interventions.
